# Can digital transformation change a firm's green innovation strategy? Evidence from China's heavily polluting industries

**DOI:** 10.1016/j.heliyon.2024.e24676

**Published:** 2024-01-20

**Authors:** Hongfei Cheng, Yuxin Li, Yaling Pang, Jing Zhao, Kui Fu

**Affiliations:** a School of Economics and Management, Shihezi University, Shihezi, 832003, China; b College of Economics and Management, Nanjing University of Aeronautics and Astronautics, Nanjing, 210016, China; c Department of Management and Engineering, Linköping University, Linköping, 58183, Sweden

**Keywords:** Digital transformation, Green technology innovation, Strategy selection, Heavily polluting enterprises, Capability-attitude, External constraint

## Abstract

Enterprises are facing the superimposed challenges of digitalization and greening. The shift from reactive green technology innovation (RGT) to proactive green technology innovation (PGT) has special significance for sustainable economic development. Which strategies will companies choose? Can digital transformation (DT) motivate companies to transform their green innovation strategies? Enterprises' green innovation strategy choices must be explained with regard to digitalization. The purpose of this paper is to reveal how digitalization affects the choice of green innovation strategies and to provide a realistic basis for the sustainable development of heavily polluting enterprises. We formulated a “DT-capability-strategy" theoretical framework incorporating external constraints and internal attitudes to empirically test the microdata of 505 heavily polluting enterprises. The results show that: (1) DT can shift the heavily polluting enterprises' green innovation strategies from RGT to PGT. Endogenous tests and robustness tests support this conclusion. (2) A mechanism test shows that environmental regulations cannot significantly regulate a green innovation strategy. Only a company's capabilities and attitudes can influence PGT but their effects on RGT are not statistically significant. (3) The influence of DT on green innovation strategies shows multi-dimensional heterogeneity in the digital infrastructure, scale, and innovation level of the enterprise. The conclusions provide implications for enterprises to integrate their digital and green behaviors.

## 1 Introduction

Global warming and environmental pollution have become important factors affecting sustainable development. How to achieve green economic and social development is one of the policy challenges faced by the world [1]. In 2020, China proposed peaking CO_2_ emissions before 2030 and achieving carbon neutrality before 2060. Enterprises' production activities, especially those of heavily polluting enterprises, are one of the important factors causing climate warming and environmental pollution. Such enterprises have always been a problem in the area of environmental governance. According to the 2021 China Ecological Environment Statistical Yearbook, the top three industries in sulfur dioxide and nitrogen oxide emissions are all heavily polluting industries. These industries accounted for 71.3 % of total sulfur dioxide emissions (industrial sources) and 82.1 % of nitrogen oxides.^1^ Therefore, environmental governance urgently needs the green transformation of heavily polluting industries. The transition to green technologies is seen as a sustainable way to achieve low carbon and low pollution [2], which requires the support of green technology innovation. Moreover, stimulation of the intrinsic initiatives of enterprises to transform from reactive green technology innovation (RGT) to proactive green technology innovation (PGT) is the key to sustainable development. With the rapid development of trends such as big data, artificial intelligence, and blockchains in information technology, the digital transformation (DT) of China's economy is booming [3]. DT tightly integrates the transformation of digital technology and the real economy at the micro-level, thus becoming a strong empowerment method for enterprises to innovate and grow [4]. This study views DT as the deep integration of information, computing, communication, and connectivity with an enterprise's production activities. In practice, DT involves the transformation of business processes and technological applications, thereby facilitating the internal integration and external expansion of old and new resources and capabilities [5].

This paper focuses on the effects of DT on enterprises' green innovation strategies. Some scholars have examined the effects of DT on green technology innovation [[6], [7], [8]] but only with regard to quantity. There is a lack of analyses from the perspectives of strategy and motivation. Chen et al. [9] studied the effects of multiple factors on PGT and RGT with regard to motivation but did not incorporate DT into their research framework. Therefore, whether DT could affect the green innovation strategies of enterprises is a key issue that we have considered by formulating a “DT-capability-strategy" theoretical framework that incorporates external constraints and internal attitudes. In addition, we applied a two-way fixed effect and moderated mediation effect models to answer the following questions: Can DT prompt heavily polluting companies to shift their strategies from RGT to PGT? What is the internal mechanism involved in this shift? We have found that DT could help firms shift to more active green innovation strategies. Such shifts have mainly been due to their own capabilities and attitudes rather than environmental regulations. These results differ from the findings of Ali et al. [10] but support the conclusion of Chen et al. [9]. Hence, internal factors play an important role in DT's influences on the green innovation strategies of enterprises.

Aimed at reducing environmental pollution, as well as saving resources and energy [11], green technology innovation encompasses a series of innovative activities related to environmental friendliness and sustainable development [12]. These activities can reduce the damage to a company's reputation caused by environmental problems [13]. The costs of environmental investments can be offset by the value generated from green technology innovation [14], which can also increase the intangible assets and values of enterprises, thereby enhancing their competitive advantages [15]. However, environmental strategies that were used to meet only environmental regulatory standards may no longer be applicable at the stage of green development [16]. Heavily polluting enterprises must shift to more proactive green innovation strategies in order to acquire strong technical guarantees for their own environmental legitimacy and to be able to seize green advantages.

In practice, green technology innovation is considered to be complex and novel while the factors or policies affecting it and an enterprise's strategic choices are believed to be complex and diverse [17]. Internal factors mainly come from the enterprise itself and include dynamic ability, organizational ability, environmental strategic orientation, environmental responsibility cognition, and green innovation intention [[18], [19], [20], [21]]. These characteristics reflect both the capabilities and intentions of the enterprise. The strengthening of its own capabilities and intentions will gradually clarify its green technology innovation strategy [22,23]. External factors include financial support and external pressure. Financing constraints are the primary obstacle that restricts green technology innovation [24,25]. Digital finance has removed the traditional finance obstacles and has provided a convenient way for enterprises to fund their innovation activities [26,27]. External pressures also involve oversight mechanisms, such as external stakeholders' supervision, government-imposed environmental regulations, and public environmental oversight [10,[28], [29], [30], [31]]. Reasonable environmental regulatory and supervisory mechanisms will produce benefits in innovation compensation, which, in turn, will encourage companies to innovate green technologies [32]. To transmit green signals (such as environmental protection) to the outside world and maintain or enhance their corporate reputations under external pressures [33], enterprises will actively engage in green technology innovation [34].

The academic community has no consensus about the effects of internal and external factors on green innovation. Few studies have integrated internal and external factors into the same framework for studying DT. Some scholars have discussed how DT has economic effects on enterprises’ innovation performance and financial performance by changing their organizational models, management efficiency, and dynamic capabilities [[35], [36], [37], [38]]. Other scholars have paid more attention to the environmental effects on the optimization of industrial structures and the utilization of non-fossil energy [[39], [40], [41], [42]]. Some scholars have concluded that DT is environmentally friendly. Conversely, other scholars have argued that the use of digital technologies (e.g., information and communication technologies) increases energy consumption [43,44]. Scholars have found that the relationship between DT and environmental performance is nonlinear [3], so it has not significantly improved environmental quality. In addition, green technology innovation is a crucial way for DT to exert green effects [6,7]. Digital technology applications are constantly changing the organizational, coordination, and environmental management perceptions of enterprises [21,37,45]. Moreover, all of these changes affect green technology innovation [8]. Information sharing has increased the degree of corporate environmental information disclosure [46], which, in turn, enables the government and the public to better play the role of overseers [47].

In summary, the research on DT and green technology innovation is relatively rich but has focused on one aspect of DT. Few scholars have considered capabilities, attitudes, and environmental regulations within a single framework. Moreover, most previous studies have regarded enterprises as passive productivity tools from a technical perspective and have investigated green technology innovation with regard to quantity. This study has focused more on self-awareness, initiative, and the interpretation of enterprises' strategies by incorporating DT and green innovation strategies into a unified framework of “DT-capability-strategy", which involves comprehensive capabilities, corporate attitudes, and external constraints. The results have important implications for exerting positive corporate environmental strategic initiatives and reveal that enterprises are compatible with digitalization and green development.

The marginal contributions of this study are threefold. (1) Incorporation of DT and the green innovation strategies into a unified framework, which was tested empirically with microdata. (2) Resource-based analysis of the internal factors (abilities and attitudes) and external constraints (environmental regulations), which enriches the research on corporate initiatives and external stakeholders. The findings may help managers and policymakers understand the digitalization and green innovation strategies of enterprises. (3) Analysis of the heterogeneous effects of DT on green innovation strategies with regard to local digital infrastructures, as well as enterprises’ scales and levels of innovation.

## 2 Theoretical basis and hypotheses

### 2.1 DT and green innovation strategies

In enterprises, DT can integrate departmental and employee functions through resource integration and reconstruction [48], as well as facilitate cross-departmental exchange and dissemination of information about environmental practices. It can also strengthen formal and informal channels for the exchange of such information [49]. According to the resource-based view [50], these changes will update or enrich a company's resource base, increase the information and knowledge reserves of green technology innovation, and enhance its green innovation capabilities. DT greatly facilitates the real-time sharing of information, which improves an enterprise's ability to capture environmental knowledge [51], understand environmental problems, enhance its perceptions of the external environment [49], and strengthen its dynamic coordination and organizational capabilities. Andriopoulos [52] argued that an organization's dynamic capability influenced its creativity. As innovation typically has a high degree of uncertainty, dynamic capability is one of its key drivers [19,53]. Organizational changes can activate the self-awareness and initiatives of enterprises to shift toward green innovation strategies [54], as well as move away from reactive to more proactive strategies. Reactive green innovation strategies that simply meet environmental regulations can no longer achieve competitive advantage or alleviate isomorphic pressures. To test this statement, we formulated our first hypothesis.

Hypothesis 1 Digital transformation (DT) motivates enterprises to shift from more reactive to more proactive green innovation strategies.

### 2.2 Mediating effect: comprehensive financial capacity

Green technology innovation is a high-risk, long-cycle (R&D cycle and income cycle), and dual-externality innovation activity [25], which is more pronounced in PGT than in RGT. From the perspectives of a resource-based view and the stakeholders, the survival of an enterprise – and whether that enterprise has sufficient resources to fulfill other responsibilities – depends on its fulfillment of economic responsibilities [55]. If a company's survival is in question, it is hard to imagine that company's investing in green innovation or adopting proactive green innovation strategies. In addition, there are unknown costs and risks associated with a shift in strategy [56]. If a company wants to change its green innovation strategy, the company must ensure that it has sufficient funds and resilience to support green technology innovation. Therefore, the comprehensive financial capabilities of an enterprise are a fundamental and necessary prerequisite for green technology innovation.

According to a cost-benefit analytical framework, the deep integration of digital technology and enterprises serves to optimize their production processes [45] and further reduce production costs. Another benefit is the potential to reduce internal control costs [57]. DT's ability to overcome resource constraints can reduce the hidden costs of resource acquisition [51]. Real-time information sharing, cross-departmental linkages, and real-time supply chain docking all serve to improve management efficiency and increase dynamic coordination capabilities [35,38], further reducing coordination costs within and outside the organization. Enterprises can increase their revenues by using information technology to better analyze and locate market opportunities, meet market demands, adapt to their external environments, and streamline their production and sales operations. The reduction of costs and expansion of revenues improve capabilities and allow enterprises to allocate more resources to green technology innovation, implement proactive innovation strategies in adverse situations and crises [58], and achieve sustainable competitive advantages. To test for these effects of DT, we formulated our second hypothesis.

Hypothesis 2 Digital transformation (DT) enhances the capabilities of enterprises to implement green innovation strategies.

### 2.3 Moderating effects: internal attitudes versus external constraints

Some scholars have argued that only internal factors could promote PGT [9]. The more sustainable a company, the more likely that company would invest in green technology innovation [59]. Positive attitudes tend to lead to proactive environmental strategies [49]. Corporate social responsibility is an important factor for sustainable development [60] because it addresses ethical issues in corporate decision-making and behaviors. It also means that businesses voluntarily integrate social and environmental issues into their operations [55]. Corporate social responsibility can also create intangible resources such as reputation and organizational culture [61], as well as strengthen the comprehensive capabilities of enterprises [62], thus helping them to develop sustainable competitive advantages. A company's prevailing internal attitudes influence its choice of environmental strategy, which further affects the direction of its capabilities. If a company pays more attention to sustainable development, it is more likely to adopt proactive environmental strategies that allocate more capacity and resources to PGT. The corporate social responsibility score is used to express the internal attitudes of a firm as an indicator of its tendencies to select green innovation strategies.

Porter held that moderate regulation produces an innovation compensation effect and incentivizes companies to engage in targeted technological change [63]. However, overly stringent regulations can significantly increase the burden on companies and limit their green innovation activities [64]. We emphasize spontaneous changes in strategy and external environmental regulations as auxiliary. As digitalization enhances an enterprise's comprehensive capabilities, its attitudes are actively adjusting its green innovation strategies. The attitudes are manifested in the enterprise's ability and willingness to proactively seize the advantages of green technology innovation, rather than passively meeting environmental regulations. To test how an enterprise's internal attitudes and the external constraints imposed by environmental regulations affect its adoption of green innovation strategies, we formulated our third hypothesis.

Hypothesis 3a Internal attitudes (corporate social responsibility) have positive effects on a firm's capability for implementing a proactive green innovation strategy.

Hypothesis 3b External constraints (environmental regulations) cannot effectively regulate the direction of a firm's capabilities or affect its proactive green innovation strategies.

**Fig. 1:**
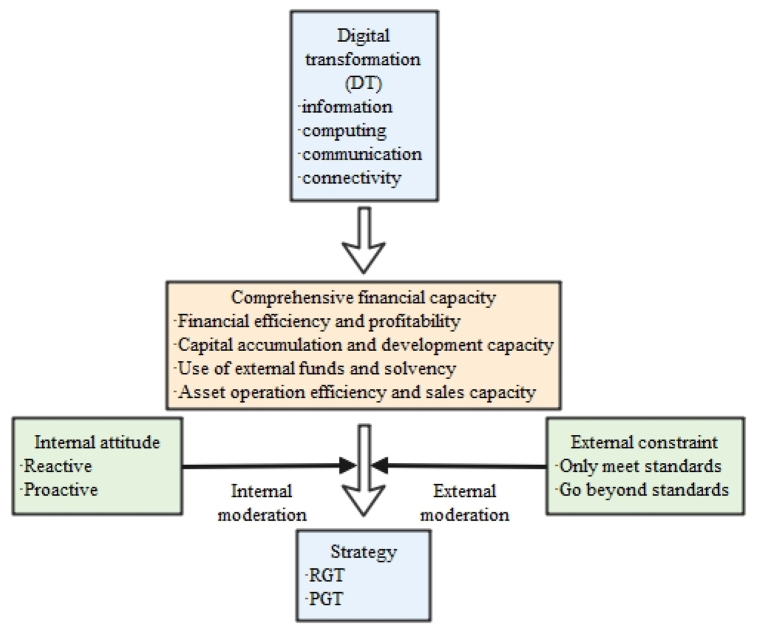
Theoretical framework.

The above discussion is summarized by the theoretical framework shown in Fig. 1.

## 3 Methodology and data

### 3.1 Model specifications

A two-way fixed effect model was applied to analyze the behaviors of the firms [3,30,45] and identify the effects of DT on their green innovation strategies. The two-way fixed effect model can eliminate the interferences of unobservable factors, reduce estimation biases, and improve the confidence of the results [65], but it requires panel data and the characteristics of big sample observations. The data in this study meet the above requirements, so it is appropriate to adopt this model. This study has also accounted for time lags in the influence of DT on green innovation strategy. The baseline regression model is:

(1) $$Y_{i,t}=\alpha +\beta DT_{i,t-1}+\gamma X_{i,t}+\lambda_{i}+\theta_{t}+\delta_{j}+\varepsilon_{i,t}$$

where *Y*_*i,t*_ is the number of green patent applications (*TGT)*, *PGT*, and *RGT*; *DT*_*i,t*-1_ is the level of digital transformation in an enterprise; *X*_*i,t*_ represents the control variables (*Scale*, *Age*, *Pid*, *TobinQ*, *Asl*, *Top10*, *Owner*, *Inv*, *Ginv*, and *ER*). The variable *i* denotes the enterprise, *t* denotes the year, and *j* represents the industry. *λ*_*i*_, *θ*_*t*_, and *δ*_*j*_ denote the fixed effects of firm, year, and industry, respectively. Finally, *ε*_*i,t*_ is the random error term.

### 3.2 Variable declarations

#### 3.2.1 Dependent variables: green innovation strategies

Since direct measurements of green innovation strategy are not easy [9], green invention and utility model patents are used to respectively express PGT and RGT, which are the proportions of the respective applications out of the total number of patent applications. The technical contents of green invention patents are quite detailed, so they can promote the technological progress of enterprises and enhance their green competitive advantages, but these results are both difficult and risky to achieve. Li and Zheng [66] argued that enterprises must implement proactive strategies to achieve breakthroughs in green invention patents. The technical contents of green utility model patents are less detailed, so they are easier to register. Hence, the motivation of enterprises in applying for such patents may only be to cater to government policies or regulations. We have studied the effects of DT on PGT and RGT to judge whether the green innovation strategies employed by enterprises have changed. For a preliminary investigation, we examined the effects of DT on TGT. We used the number of patent applications instead of the number of patents granted because patent applications reflected the strategic choices and innovation dynamics of enterprises more directly. External factors, such as administrative approval, cannot interfere with these characteristics, which, therefore, represent the autonomy of the enterprises.

#### 3.2.2 Independent variable: DT

So far, no unified standard exists for measuring DT. In the literature [67], it is usually measured by “digital"-related word frequencies in the “management discussion and analysis" (MD&A) sections of firms' annual reports because the qualitative descriptions in these sections indicate, at least partially, both the companies’ recent developments and their plans for future development [68,69]. First, the “lexicon" is used to filter specific texts for building a “digitized" vocabulary. Second, the contents of the MD&A sections are extracted, and then the jieba word segmentation tool in Python is employed to segment the contents and propose stop words. Finally, the word frequencies and their proportions for “digitalization" are used to measure DT.

**Fig. 2:**
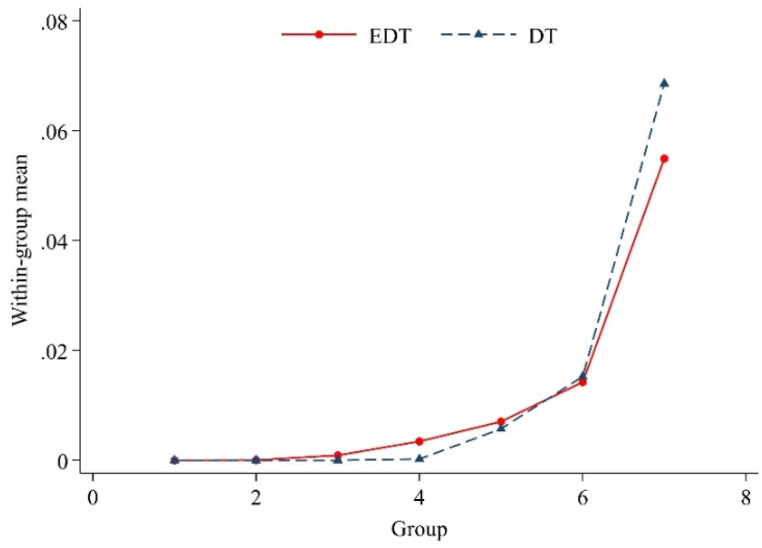
Digital steering index inspection.

The basic assumption of this measurement method is that the contents of the annual reports are based on objective statements regarding the companies' actual operations. The relevant words can also better reflect the companies' degree of digitalization. Therefore, the enterprises’ digitalization-related intangible assets are also identified to test the credibility of the DT indicators. In Song et al. [70], when an intangible asset line item contains keywords related to digital technology, the line item is marked as “digitalization-related". Then, the economic digitalization (EDT) is expressed by the proportion of “digitalization-related" intangible assets. Next, EDT and DT are divided into seven groups, and then the intra-group means are obtained and compared, as shown in Fig. 2. The degree of DT measured by text analysis is basically consistent with the digital intangible asset indicators, thereby indicating that the DT indicators measured in our study have credibility.

#### 3.2.3 Mediator variable: comprehensive financial capability (M)

The comprehensive financial capacity of an enterprise contains four capabilities and involves four types of indicators.

(1) Financial efficiency and profitability: return on net assets
(2) Capital accumulation and development capacity: capital accumulation rate
(3) Use of external funds and solvency: asset-liability ratio
(4) Asset operation efficiency and sales capacity: total asset turnover

#### 3.2.4 Moderator variables: external constraints (W_1_) and internal attitudes (W_2_)

External constraints measure the constraints and guidance of the external environment on enterprises’ development. We used local environmental regulations to represent the external constraints, which are determined by the frequencies of environmental protection words such as “energy consumption", “low carbon", “environmental protection", “green", “PM2.5″, “PM10″, “ecology", “emission reduction", “carbon dioxide", “sulfur dioxide", “chemical oxygen demand", and “pollution". Internal attitudes measure whether and to what extent companies are willing to integrate social and environmental issues into their corporate environmental strategies. As per Li et al. [71], we used the corporate social responsibility score published by Hexun.com to quantify the internal attitudes.

#### 3.2.5 Control variables

To exclude other factors [20,21], the following variables were controlled: enterprise scale (Scale), which is expressed as the total assets of a company; age of the enterprise (Age), which is expressed as the difference between the current year and the year of the enterprise's establishment. Other variables include percentage of independent directors (Pid), Tobin's Q (TobinQ), gearing ratio (Asl), and equity concentration (Top10), which refers to the proportion of shareholding held by the top 10 shareholders. The nature of equity (Owner) refers to whether an enterprise is state-owned (1) or not (0). Innovation capacity (Inv) is expressed by the number of patent applications. Green innovation level (Ginv) is the number of green patent applications. Environmental regulation (ER) is measured in the same way as external constraints (W_1_).

### 3.3 Data sources

The DT data come from the annual reports of companies, the environmental regulation data come from government work reports, the corporate social responsibility data come from Hexun.com, the basic information and the financial data come from the China Stock Market & Accounting Research Database, and the patent application data come from the Chinese Research Data Services Platform. The following steps were implemented: (1) exclusion of ST, *ST, and other enterprises in special states; (2) exclusion of enterprises that are not in normal transactions; (3) exclusion of enterprises with many missing data; (4) exclusion of companies listed after 2011. The number of observations obtained was 4545. Table 1 shows the descriptive statistics for each variable.

**Table 1:** Descriptive statistics of variables.

Variables	Obs.	Mean	SD	Min.	Max.
TGT	4545	0.072	0.208	0.000	1.000
PGT	4545	0.044	0.153	0.000	1.000
RGT	4545	0.028	0.118	0.000	1.000
DT	4545	0.013	0.034	0.000	0.230
Scale (log)	4545	22.582	1.335	16.117	27.099
Age (log)	4545	2.828	0.352	0.693	3.714
Pid	4544	0.369	0.052	0.231	0.667
TobinQ (log)	4545	0.501	0.500	−0.358	3.343
Asl	4545	0.476	0.270	0.007	10.082
Top10	4544	0.495	0.213	0.107	0.985
Owner	4545	0.309	0.462	0.000	1.000
Inv (log)	4545	1.190	1.491	0.000	6.903
Ginv (log)	4545	0.234	0.663	0.000	5.283
ER	4545	0.595	0.208	0.025	1.815
M	4545	0.076	0.044	0.006	0.621
W_1_	4545	0.595	0.208	0.025	1.815
W_2_	4545	4.529	3.853	−15.000	23.000

## 4 Results

### 4.1 Baseline regression

Based on Eq. (1), we conduct an empirical study on the effect of DT on green innovation strategies of heavily polluting enterprises. The baseline regression results are shown in Table 2. The regression controls for individual and temporal effects. In Column (1), the coefficient is equal to 0.058 for the effect of DT on TGT, but this value is not statistically significant. This result leads us to consider if there has been a shift in green innovation strategies within the enterprises, who have been prompted to reallocate green innovation resources. Therefore, the focus shifts to the changes in PGT and RGT. The effects of DT on PGT and RGT are 0.156 and −0.098, respectively, which are significant at the 5 % level. For each percentage increase in the DT of the enterprises, there is a corresponding increase in PGT by 15.6 % and a corresponding decrease in RGT by 9.8 % when other conditions remain unchanged. Hence, we can conclude that DT has led to changes in the green innovation strategies of heavily polluting enterprises, which are shifting from RGT to PGT. DT's promoting effect on PGT is stronger than its inhibiting effect on RGT. The shift in strategy has changed the enterprises' allocation of resources by increasing the resource inputs of PGT and reducing the resource inputs of RGT. This result supports Hypothesis 1.

**Table 2:** Benchmark regression results.

	(1)	(2)	(3)	(4)	(5)
	TGT	PGT	RGT	PGT	RGT
L.DT	0.058	0.156**	−0.098**	0.156**	−0.098**
	(1.18)	(1.99)	(-2.40)	(1.99)	(-2.40)
Control variables	Yes	Yes	Yes	Yes	Yes
Year FE	Yes	Yes	Yes	Yes	Yes
Company FE	Yes	Yes	Yes	Yes	Yes
Industry FE	Yes	No	No	Yes	Yes
Observations	4039	4039	4039	4039	4039
*R*-squared	0.560	0.484	0.420	0.484	0.420

Note: L. denotes a time lag; t-statistics are in parentheses; ***p < 0.01, **p < 0.05, and * *p* < 0.1; estimates calculated with clustered standard errors.

### 4.2 Robustness tests

#### 4.2.1 Endogenous treatment

Relative indicators were used in the baseline regression while the fixed effects were controlled to mitigate endogenous problems, but there may still be factors beyond control that could cause endogenous problems. Therefore, we used the following methods to alleviate potential endogenous problems.

(1) Propensity score matching (PSM) method

**Fig. 3:**
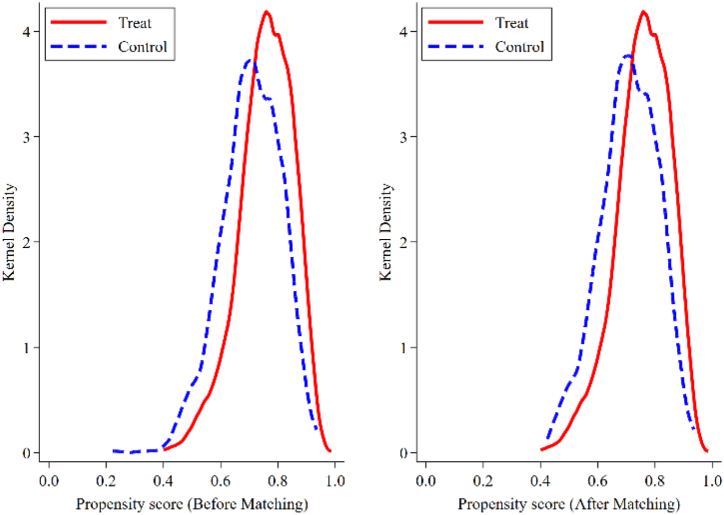
Propensity score and kernel density maps.

The PSM method can alleviate the selection problem and effectively control for sample selectivity bias. The method's basic idea is to generate matching scores that are based on certain characteristics. Then, new groups based on the matching scores are established. This study classified enterprises that had not digitized into control groups and assigned them a value of 0. Enterprises that had undergone DT were assigned into processing groups and given a value of 1. The two groups were matched with K-nearest-neighbors matching within caliper. Fig. 3 shows the changes in kernel density before and after matching. As can be seen from Fig. 3, Observations that do not meet the matching characteristics have been excluded, which reduces the sample selectivity bias. Table 3 shows the PSM regression results. The effects of DT on PGT and RGT are 0.149 and −0.099, respectively, which are significant at the levels of 10 % and 5 %, respectively. The regression results are also significant because they still support the benchmark regressions after controlling the sample self-selection bias.

(2) Instrumental variable (IV) method

**Table 3:** Endogenous test results.

	PSM			IV		
	(1)	(2)	(3)	(4)	(5)	(6)
	TGT	PGT	RGT	TGT	PGT	RGT
L.DT	0.051	0.149*	−0.099**	0.186	0.294*	−0.108***
	(1.06)	(1.94)	(-2.38)	(1.22)	(1.88)	(-3.14)
Control variables	Yes	Yes	Yes	Yes	Yes	Yes
Year FE	Yes	Yes	Yes	Yes	Yes	Yes
Company FE	Yes	Yes	Yes	Yes	Yes	Yes
Industry FE	Yes	Yes	Yes	Yes	Yes	Yes
Under-identification test				3.068*	3.068*	3.068*
Weak-identification test				1033.6	1033.6	1033.6
Observations	4002	4002	4002	4039	4039	4039
*R*-squared	0.561	0.484	0.421	0.151	0.114	0.068

Note: L. denotes a time lag; t-statistics are in parentheses; ***p < 0.01, **p < 0.05, and * *p* < 0.1; estimates calculated with clustered standard errors.

Implicit variables such as macro-policy shocks (smart city pilot policies) may have simultaneous effects on DT and green technology innovation; which would create endogenous problems. Referring to Lewbel [72], we formulated an internally valid instrumental variable to alleviate such problems by calculating the mean DT by two digits that represent the industry and province, as well as computing the cubic of the difference between DT and the mean. Table 3 also shows that the effects of DT on PGT and RGT are 0.294 and −0.108, respectively, which are significant at the levels of 10 % and 1 %, respectively. The under-identification test and weak-identification test verified the instrumental variable to be reliable. The coefficients fluctuate to a certain extent but still support the benchmark regression results in the direction of the influence.

#### 4.2.2 Robustness test

(1) Replacement of dependent variables

We verified the robustness of the results by substituting the dependent variables. The replaced PGT (SPGT) and replaced RGT (SRGT) are relative to the proportion of green patent applications. Table 4 shows that the coefficients are equal to 0.300 and −0.259 for the effects of DT on SPGT and SRGT, respectively. The results do not change qualitatively and clearly indicate that the basic conclusions are robust and credible.

(2) Elimination of zero-patent enterprises

**Table 4:** Robustness checks of baseline regression.

	Replace dependent variables		Eliminate zero-patent enterprises		Dynamic panel data model	
	(1)	(2)	(3)	(4)	(5)	(6)
	SPGT	SRGT	PGT	RGT	PGT	RGT
L.DT	0.300*	−0.259*	0.115**	−0.095**	0.151**	−0.096**
	(1.83)	(-1.85)	(2.10)	(-2.39)	(2.03)	(-2.54)
L.PGT					0.128*	
					(1.73)	
L.RGT						0.174**
						(2.55)
Control variables	Yes	Yes	Yes	Yes	Yes	Yes
Year FE	Yes	Yes	Yes	Yes	Yes	Yes
Company FE	Yes	Yes	Yes	Yes	Yes	Yes
Industry FE	Yes	Yes	Yes	Yes	Yes	Yes
Observations	4039	4039	3072	3072	3534	3534
*R*-squared	0.508	0.251	0.471	0.411		
AR (1)					Z = −4.87	Z = −5.88
AR (2)					Z = 0.94	Z = 0.82
Hansen test					14.58	17.20

Note: L. denotes a time lag; t-statistics are in parentheses; ***p < 0.01, **p < 0.05, and * *p* < 0.1; estimates calculated with clustered standard errors (dynamic panel data model).

Some of the companies have never applied for a patent (zero-patent) during the sample period, so these samples may have interfered with the regression. Referring to Song et al. [70], we excluded the zero-patent enterprises for regression, as also shown in Table 4. The effects of DT on PGT and RGT are 0.115 and −0.095, respectively. The results further verify the conclusion that the green innovation strategies of the heavily polluting enterprises changed after DT.

(3) Dynamic panel data model

To exclude the influences of past innovation behaviors on current innovation strategies, we used the dynamic panel data model to test the robustness. Columns (5)–(6) in Table 4 show that the PGT and RGT of the first-order lag are significant at the levels of 10 % and 5 %, respectively. The effects of DT on PGT and RGT are 0.151 and −0.096, respectively, which are significant at the 5 % level. The results are basically consistent with the benchmark regression and indicate that the baseline estimates are robust.

### 4.3 Heterogeneity analysis

#### 4.3.1 Heterogeneity in different digital infrastructures

DT relies on local digital infrastructures, so different digital infrastructures may produce different results. Since 2013, the Chinese government has announced three batches of broadband pilot cities to strengthen their network infrastructures. Therefore, we divided the samples into non-pilot areas and pilot areas for regression (see Table 5). Note that DT promotes the PGT (0.264) of the pilot areas but reduces the RGT (−0.140). For the non-pilot areas, DT reduces the RGT (−0.049) but does not significantly affect the PGT (−0.118). These results show that in the pilot cities, for each percentage increase in the DT of the enterprises is a corresponding increase in PGT by 26.4 % and a corresponding decrease in RGT by 14 %. The effects of DT on the green innovation strategies are obvious only for the enterprises of the pilot cities. The heterogeneity analysis shows that relatively complete digital infrastructures are more conducive to the DT of the enterprises. Consequently, the DT can more easily affect their green innovation strategies.

**Table 5:** Heterogeneity tests of different digital infrastructures.

	Non-pilot areas		Pilot areas	
	(1)	(2)	(3)	(4)
	PGT	RGT	PGT	RGT
L.DT	−0.118	−0.049*	0.264*	−0.140**
	(-0.71)	(-1.92)	(1.95)	(-2.34)
Control variables	Yes	Yes	Yes	Yes
Year FE	Yes	Yes	Yes	Yes
Company FE	Yes	Yes	Yes	Yes
Industry FE	Yes	Yes	Yes	Yes
Observations	1456	1456	2583	2583
*R*-squared	0.447	0.391	0.512	0.446

Note: L. denotes a time lag; t-statistics are in parentheses; ***p < 0.01, **p < 0.05, and * *p* < 0.1; estimates calculated with clustered standard errors.

#### 4.3.2 Heterogeneity at different scales

Enterprises of different sizes may differ in their resource bases and organizational flexibility, so DT may have different effects on their green innovation strategies. We divided the enterprises into large-scale and small-scale for regression (see Table 6). The results illustrate that for the small-scale enterprises, DT reduces RGT (−0.114) but does not significantly affect PGT (0.078). For the large-scale enterprises, DT increases PGT (0.316) which is significant at the level of 1 %. The effect is significantly greater than that of the whole sample regression (0.316 > 0.156) but not significant for RGT (−0.048).

**Table 6:** Heterogeneity at different scales.

	Small scale		Large scale	
	(1)	(2)	(3)	(4)
	PGT	RGT	PGT	RGT
L.DT	0.078	−0.114**	0.316***	−0.048
	(0.51)	(-1.99)	(2.69)	(-0.82)
Control variables	Yes	Yes	Yes	Yes
Year FE	Yes	Yes	Yes	Yes
Company FE	Yes	Yes	Yes	Yes
Industry FE	Yes	Yes	Yes	Yes
Observations	2004	2004	2004	2004
*R*-squared	0.525	0.331	0.475	0.456

Note: L. denotes a time lag; t-statistics are in parentheses; ***p < 0.01, **p < 0.05, and * *p* < 0.1; estimates calculated with clustered standard errors.

On the whole, large-scale enterprises have resource advantages, so DT improves their performance and resource allocation efficiency. In addition, DT enhances their ability to bear risks and to innovate, which enable them to increase their PGT without reducing RGT. Conversely, small-scale enterprises have relatively scarce resources and are burdened with weak bargaining power relative to the upstream and downstream enterprises. Their overall ability to resist risks is not strong. After DT, small enterprises cannot quickly shift to PGT because they need some time to adjust and reallocate their limited resources toward innovation.

#### 4.3.3 Heterogeneity at different levels of innovation

The sample was divided into higher and lower levels of innovation. Table 7 shows that for the high-level enterprises, the effects of DT on PGT are not significant (0.073) and the coefficient is equal to −0.174 for the effects of DT on RGT. Undoubtedly, for low-level enterprises, DT promotes the shifts in green innovation strategies from RGT to PGT, whose coefficients are −0.097 and 0.203, respectively. Companies with different levels of innovation also have different technological resources. To obtain green competitive advantages, high-level enterprises are more likely to turn to proactive green innovation strategies. DT only serves to strengthen the existing innovation system and the marginal effects on strategy transformation are not obvious. Low-level enterprises have weak technical foundations and relatively scarce innovation resources. Unlike high-level enterprises, they are unable to chase green competitive advantages in the same period, so temporal and spatial differences appear in their innovation strategies. After DT, low-level enterprises experience more obvious marginal effects that strengthen their technical foundations and enrich their innovation resources, thus improving their abilities to bear innovation risks and adopt more proactive green innovation strategies.

**Table 7:** Heterogeneity at different levels of innovation.

	Low level		High level	
	(1)	(2)	(3)	(4)
	PGT	RGT	PGT	RGT
L.DT	0.203**	−0.097***	0.073	−0.174**
	(2.05)	(-2.89)	(1.02)	(-2.37)
Control variables	Yes	Yes	Yes	Yes
Year FE	Yes	Yes	Yes	Yes
Company FE	Yes	Yes	Yes	Yes
Industry FE	Yes	Yes	Yes	Yes
Observations	2002	2002	1945	1945
*R*-squared	0.397	0.229	0.673	0.620

Note: L. denotes a time lag; t-statistics are in parentheses; ***p < 0.01, **p < 0.05, and * *p* < 0.1; estimates calculated with clustered standard errors.

### 4.4 Test of mechanisms

The benchmark regression shows that DT has prompted heavily polluting enterprises to change their green innovation strategies, but what are the channels and boundary conditions for this change? To find the answer, we formulated a mediating effect model with the moderating effects of the firms’ capabilities, internal attitudes, and external constraints. As there are time lags, these variables are treated with delays of one period. The interaction items of the mediator variable and moderator variables are treated with lags of one period:

(2) $$M_{i,t}=\alpha +\beta DT_{i,t-1}+\gamma X_{i,t}+\lambda_{i}+\theta_{t}+\delta_{j}+\varepsilon_{i,t}$$

(3) $$Y_{i,t}=\alpha +\beta_{1}DT_{i,t-1}+\beta_{2}M_{i,t-1}+\gamma X_{i,t}+\lambda_{i}+\theta_{t}+\delta_{j}+\varepsilon_{i,t}$$

(4) $$Y_{i,t}=\alpha +\beta_{1}DT_{i,t-1}+\beta_{2}M_{i,t-1}+\beta_{3}W_{i,t-1}+\beta_{4}MW_{i,t-1}+\gamma X_{i,t}+\lambda_{i}+\theta_{t}+\delta_{j}+\varepsilon_{i,t}$$

where *Y*_*i,t*_ is *PGT* and *RGT*; *DT*_*i,t*-1_ is the level of digital transformation; *M* represents the mediation variable, i.e., the firm capabilities; *W* represents the moderation variables, which are environmental regulations (external constraints *W*_1_) and corporate social responsibility (internal attitudes *W*_2_); *MW* represents the interaction between *M* and *W*; *X*_*i,t*_ represents the control variables (*Scale*, *Age*, *Pid*, *TobinQ*, *Asl*, *Top10*, *Owner*, *Inv*, *Ginv*, and *ER*). The variable *i* denotes the enterprise, *t* denotes the year, and *j* represents the industry. *λ*_*i*_, *θ*_*t*_, and *δ*_*j*_ denote the fixed effects of firm, year, and industry, respectively. Finally, *ε*_*i,t*_ is the random error term.

#### 4.4.1 Tests of mediating and moderating mechanisms (proactive strategies)

Based on Eqs. (2), (3), (4), we test the mechanism of DT on proactive strategies of heavily polluting enterprises. Table 8 reports the channel and boundary conditions under which DT affects PGT. Columns (1)–(4) show that the coefficient is equal to 0.072 for the effects of DT on comprehensive capabilities (M). For each percentage increase in DT, comprehensive capabilities increase by 7.2 %. The mediating effect of M is 0.272 and significant at the 1 % level. The Sobel test supports the mediation effect at a significance level of 1 %. This finding shows that DT promotes the comprehensive capabilities of the enterprises so that they have the abilities and resources to transform their green innovation strategies. This conclusion validates Hypothesis 2.

**Table 8:** Results of the mechanism tests (Proactive strategies).

	(1)	(2)	(3)	(4)	(5)	(6)
	PGT	M	PGT	PGT	PGT	PGT
L.DT	0.156**	0.072**		0.152*	0.154*	0.151*
	(1.99)	(2.57)		(1.91)	(1.92)	(1.93)
L.M			0.276***	0.272***	0.218	0.200*
			(2.62)	(2.58)	(1.40)	(1.79)
L.W_1_					−0.003	
					(-0.13)	
L.MW_1_					0.112	
					(0.58)	
L.W_2_						−0.002
						(-1.31)
L.MW_2_						0.022*
						(1.80)
Control variables	Yes	Yes	Yes	Yes	Yes	Yes
Year FE	Yes	Yes	Yes	Yes	Yes	Yes
Company FE	Yes	Yes	Yes	Yes	Yes	Yes
Industry FE	Yes	Yes	Yes	Yes	Yes	Yes
Observations	4039	4039	4039	4039	4039	4039
*R*-squared	0.484	0.220	0.484	0.485	0.485	0.485
Sobel test		Z = 2.096				

Note: L. denotes a time lag; t-statistics are in parentheses; ***p < 0.01, **p < 0.05, and * *p* < 0.1; estimates calculated with clustered standard errors.

Column (5) shows that the influence of MW_1_ on DT is 0.112, but the result is not significant. As external constraints, environmental regulations (W_1_) do not effectively regulate the direction of the comprehensive capabilities (M) and do not promote PGT. This conclusion validates Hypothesis 3b.

**Fig. 4:**
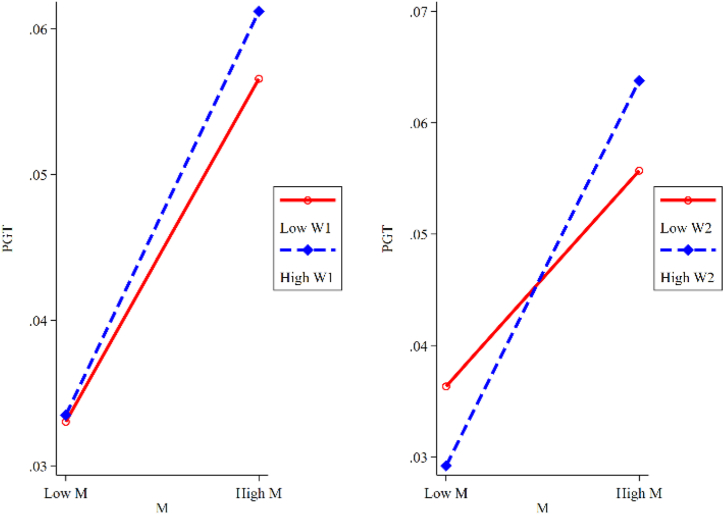
Moderating effects of external constraints and internal attitudes (Proactive strategies).

Column (6) shows that the coefficient is equal to 0.022 for the effect of MW_2_ on PGT and is significant at 10 % level. The result is a significant positive value, which indicates that the internal attitudes (W_2_) toward green and sustainable development will positively adjust the direction of the comprehensive capabilities (M) and strengthen the positive effects of DT on PGT. This conclusion validates Hypothesis 3a. Fig. 4 shows the moderating effects of W_1_ and W_2_ on M, demonstrating that W_2_ can effectively moderate the mediating effect of M on PGT.

In summary, an enterprise's internal factors of ability and attitude play intermediary and regulatory roles, which positively affect the enterprise's positive green innovation strategy. As external constraints, environmental regulations cannot effectively moderate the influence channels of the enterprise's capabilities and thus the regulations fail to promote PGT. To some extent, this result supports the conclusion of Chen et al. [9] that only positive internal factors could promote PGT. This result is an empirical verification of digitalization that contributes to the current literature.

#### 4.4.2 Tests of mediating and moderating mechanisms (reactive strategies)

Then, based on Eqs. (2), (3), (4), we test the mechanism of DT on reactive strategies of heavily polluting enterprises. Table 9 reports the effects of M, W_1_, and W_2_ on RGT for a comparison of the shifts in green innovation strategies. Columns (1)–(4) show that the effects of DT on the comprehensive capabilities (M) of the enterprises are 0.072. However, comprehensive capabilities (M) are not an effective intermediary through which DT can affect RGT. The Sobel test shows that M could not effectively mediate the process and suggests that the moderating effect's actions on the mediating effect were ineffective, i.e., neither external constraints nor internal attitudes significantly modulated the influence of DT on RGT.

**Table 9:** Results of the mechanism tests (Reactive strategies).

	(1)	(2)	(3)	(4)	(5)	(6)
	RGT	M	RGT	RGT	RGT	RGT
L.DT	−0.098**	0.072**		−0.099**	−0.101**	−0.099**
	(-2.40)	(2.57)		(-2.43)	(-2.45)	(-2.44)
L.M			−0.020	0.031	0.154	0.030
			(-0.59)	(0.38)	(1.38)	(0.28)
L.W_1_					0.024**	
					(2.22)	
L.MW_1_					−0.244*	
					(-1.69)	
L.W_2_						0.001
						(1.00)
L.MW_2_						−0.001
						(-0.05)
Control variables	Yes	Yes	Yes	Yes	Yes	Yes
Year FE	Yes	Yes	Yes	Yes	Yes	Yes
Company FE	Yes	Yes	Yes	Yes	Yes	Yes
Industry FE	Yes	Yes	Yes	Yes	Yes	Yes
Observations	4039	4039	4039	4039	4039	4039
*R*-squared	0.420	0.220	0.120	0.420	0.420	0.420
Sobel test		Z = −0.420				

Note: L. denotes a time lag; t-statistics are in parentheses; ***p < 0.01, **p < 0.05, and * *p* < 0.1; estimates calculated with clustered standard errors.

## 5 Discussions and implications

### 5.1 Discussion

Currently, heavily polluting enterprises are in a period of overlapping digital transformation (DT) and green transformation. DT is important in empowering enterprises to innovate and grow. Yin and Yu [73] pointed out that digital technology affects the organizational management and environmental awareness of enterprises. In addition, green technology innovation is a crucial way for DT to exert green effects [6,7], but the factors influencing companies to shift their green innovation strategies are complex. At present, there is insufficient research on DT and green innovation strategies, so enterprises’ strategy choices must be explained in terms of DT with regard to both theoretical and empirical aspects. This study constructed a framework that includes internal factors and external constraints. The empirical findings show that DT has led enterprises to shift from reactive green technology (RGT) to proactive green technology (PGT) innovation. The possible causes for such shifts are changes in corporate capabilities and attitudes that lead to the reallocation of resources. Wei and Sun [48] pointed out that DT promoted the restructuring of internal resources and the integration of external resources. Their conclusion is logically consistent with the conclusion of this paper.

At this stage, environmental regulations with regard to DT cannot effectively regulate green innovation strategy and the shift in strategy still depends on internal factors, such as self-awareness and attitude toward green transformation and sustainable development [[18], [19], [20], [21]]. Chen et al. [9] have shown that internal factors helped stimulate corporate autonomy. The conclusion of our study indicates that an enterprise's internal factors of abilities and attitudes play intermediary and regulatory roles, both of which positively affect the enterprise's positive green innovation strategy. As external constraints, environmental regulations cannot effectively regulate the influence channels of enterprises' capabilities and thus the regulations fail to promote PGT. To some extent, this result supports the conclusion of Chen et al. [9] that only positive internal factors can promote PGT. In addition, DT helps activate initiatives within the enterprise. Our results empirically verify the effects of digitalization and contribute to the current literature. The conclusions are of great significance for promoting the development of enterprise digitalization and greening, as well as for the formulation of relevant government policies.

### 5.2 Managerial implications

Enterprises themselves should actively embrace DT while the window of opportunity is still open. However, enterprises should fully consider their own scale, innovation conditions, and technical foundations before implementing DT. Digital technology should be used to promote an enterprise's internal management and production activities, increase information exchanges between internal and external non-stakeholders (citizens, environmental protection organizations, etc.), strengthen cross-departmental information linkages, and enhance environmental perceptions and awareness throughout the enterprise from subordinates to managers. Then, enterprises should effectively leverage the technical effects and green effects of digitalization to achieve sustainable competitive advantages.

### 5.3 Policy implications

First, DT can unquestionably stimulate the initiatives of enterprises and promote PGT. Therefore, digitalization does not conflict with greening but is compatible. The government should do a good job in its role as a public service provider. Efforts should be made to improve digital facilities, such as introducing and improving industrial internet connections and big data centers. In addition, the government should encourage enterprises to actively integrate digital technology into all aspects of production in order to empower corporate environmental governance and sustainable development through digitalization.

Second, government departments should appropriately adjust external constraints, such as environmental regulations, with a view to stimulating enterprises’ internal enthusiasm. Only internal factors, such as corporate capabilities and attitudes, can effectively promote PGT, whereas environmental regulations cannot. Therefore, governments and environmental protection authorities should carefully design environmental regulatory policies and avoid improper regulatory policies, which increase the burden on enterprises.

Third, the government should think about how to improve the comprehensive capabilities of enterprises, change their attitudes toward environmental governance, and stimulate their autonomy and initiative. For example, tax incentives, financial subsidies, and other incentives could be implemented to improve their ability to bear risks. In addition, governments should design differentiated combinations of incentives for enterprises with low levels of innovation so as to increase the enterprises’ policy preferences and motivate them to implement DT. By improving their degree of digitalization, such enterprises can activate their own positive initiatives and shift from RGT to PGT.

### 5.4 Limitations and future research

Despite the contributions of this work, refinements are required for future research. First, the inclusion of only Chinese heavily polluting companies limits the generalizability of our findings. Future research could be extended to other developing countries and other industries to complement the findings here. Second, this paper focuses on the companies’ internal mechanisms in terms of capabilities, attitudes, and external constraints. In fact, there may be other factors. As data permits, future research could explore how managers' capabilities and perceptions influence green decision-making with regard to digitalization. Finally, future research could combine analysis of both macro and micro factors, and focus on the role of digital technology in industrial structure upgrading.

## Funding statement

This research was funded by the Philosophy and Social Science Foundation of China (22AJY005) and funded by 2023 10.13039/501100009967Xinjiang Production and Construction Corps (10.13039/501100009967XPCC) Graduate Innovation Project (2023).

## Data availability statement

The data presented in this study are available on request from the corresponding author.

## CRediT authorship contribution statement

**Hongfei Cheng:** Writing – original draft, Writing – review & editing, Methodology, Funding acquisition, Formal analysis, Data curation, Conceptualization. **Yuxin Li:** Writing – review & editing, Validation, Supervision, Project administration, Methodology, Funding acquisition, Formal analysis, Conceptualization. **Yaling Pang:** Validation, Software, Methodology, Data curation, Conceptualization. **Jing Zhao:** Software, Methodology, Data curation, Conceptualization. **Kui Fu:** Writing – review & editing, Methodology, Formal analysis, Conceptualization.

## Declaration of competing interest

The authors declare that they have no known competing financial interests or personal relationships that could have appeared to influence the work reported in this paper.

## Footnotes

1 Data source: W020230118392178258531.pdf (mee.gov.cn).
